# Construct Validity and Responsiveness of the COVID-19 Yorkshire Rehabilitation Scale (C19-YRS) in a Cohort of Italian Hospitalized COVID-19 Patients

**DOI:** 10.3390/ijerph19116696

**Published:** 2022-05-30

**Authors:** Sofia Straudi, Fabio Manfredini, Andrea Baroni, Giada Milani, Giulia Fregna, Nicola Schincaglia, Riccardo Androni, Antonella Occhi, Manoj Sivan, Nicola Lamberti

**Affiliations:** 1 Department of Neuroscience and Rehabilitation, Ferrara University Hospital, 44124 Ferrara, Italy; fabio.manfredini@unife.it (F.M.); brnndr3@unife.it (A.B.); giada.milani@unife.it (G.M.); nicol.schincaglia@edu.unife.it (N.S.); a.occhi@ospfe.it (A.O.); 2 Department of Neuroscience and Rehabilitation, University of Ferrara, 44121 Ferrara, Italy; nicola.lamberti@unife.it; 3 Doctoral Program in Translational Neurosciences and Neurotechnologies, Ferrara University, 44121 Ferrara, Italy; giulia.fregna@unife.it; 4 School of Physiotherapy, Ferrara University, 44121 Ferrara, Italy; riccardo.androni@edu.unife.it; 5 Academic Department of Rehabilitation Medicine, University of Leeds, D Floor, Martin Wing, Leeds General Infirmary, Leeds LS1 3EX, UK; m.sivan@leeds.ac.uk

**Keywords:** Post-COVID Syndrome, outcome measures, rehabilitation, COVID-19

## Abstract

Post-COVID Syndrome (PCS) is characterized by physical, psychological and cognitive symptoms with a negative impact on daily activities. This study tested the responsiveness and construct validity of the original version of the COVID-19 Yorkshire Rehabilitation Scale (C19-YRS) in a cohort of Italian hospitalized COVID-19 patients. This longitudinal study involved 79 hospitalized COVID-19 patients with rehabilitation needs, assessed after 12 and 26 weeks post-infection. Functional and patient-reported outcome measures were correlated with 10 items of the C19-YRS to test the construct validity, whereas distribution-based methods were used for the responsiveness over time. After 12 weeks since infection, 88.5% of patients reported fatigue, 83.3% breathlessness, 69.4% anxiety, 55.6% depression, and 44.9% pain. Moreover, 84.6% experienced reduced mobility, 75.7% had difficulties with usual activities, and 47.4% with personal care. Single items for each symptom had significant correlation (rho 0.25–0.60) with well-established scales for these symptoms. At 26 weeks, except for anxiety, all the PCS symptoms were still present but significantly reduced. The C19-YRS had moderate responsiveness for the most represented deficit reported. The C19-YRS was a valuable patient-reported outcome for screening, assessing severity, and monitoring the persistence of symptoms after 12 and 26 weeks from SARS-CoV2 infection in a cohort of Italian hospitalized patients.

## 1. Introduction

Since the pandemic outbreak in March 2020 [1], more than 11 million people in Italy have been infected by SARS-CoV2. In China, about 14% of patients needed hospitalization and 5% intensive care management [2], with a significant burden on the public healthcare system. Most hospitalized patients needed respiratory and motor rehabilitation in acute and subacute settings to regain mobility and independence [3,4]. Moreover, it has been rapidly estimated that several symptoms and limitations can exceed the 12 weeks with a negative impact on Health-related Quality of Life (HRQoL) [5], leading to the identification of Post-COVID Syndrome (PCS). So far, more than 50 long-term effects have been classified [6]; even though the most common signs and symptoms reported are both physical, such as dyspnea, fatigue, pain, and psychological, such as anxiety and depression [7,8,9]. These symptoms can be combined in different ways, fluctuating over time, and lead to an overall impairment of mobility, reduced independence in everyday life activities and Health-Related Quality of Life (HR-QoL). The pathophysiology of PCS is still largely unknown. A combination of factors related to the pre-existing conditions (i.e., comorbidities), the acute phase (i.e., post-critical illness), and immunologic and inflammatory damage have been hypothesized [10,11]. The risk factors for developing PCS are older age, female sex, and body mass index [12]; interestingly, PCS was not influenced by COVID-19 severity [13].

A multidisciplinary assessment of PCS is warranted to guide a tailored rehabilitation and promote recovery [14,15,16]. The COVID-19 Yorkshire Rehabilitation Scale (C19-YRS), developed in the UK by Sivan et al., was the first validated patient-reported outcome measure that encompasses all domains of the WHO International Classification of Functioning, Disability and Health (ICF) [17,18,19]. It was based on symptoms and functional difficulties that were reported by COVID-19 survivors across the clinical sites in the Yorkshire region [9] and it represented a condition-specific measure for PCS. A clinician-administered, patient-reported, and digital version is now available for the C19-YRS [18]. This scale was recommended by the National Institute for Health and Care Excellence (NICE) and the England National Health Service (NHS) to assess PCS [20,21], and adopted by the WHO for their PCS self-management guide [22]. In this context, evaluating psychometrics properties in different PCS populations is mandatory to prove the usability of this outcome measure in clinical settings. This study aimed to determine the construct validity and responsiveness of using 10 items short of the C19-YRS. The hypothesis is that it can be a reliable tool to screen adults for PCS after 12 weeks from SARS-CoV2 infection and detect variations over time (26 weeks).

## 2. Materials and Methods

This was a longitudinal observational study. The protocol was approved by the Ethical Committee (EM66-2022_539/2020/Oss/AOUFe_EM1) and prospectively registered on Clinicaltrial.gov (NCT04615390). Patients with rehabilitation needs during their hospitalization stay (between January and April 2021) at Ferrara University Hospital for SARS-CoV2 infection were enrolled in this study and assessed after 12 and 26 weeks. We included men and women aged over 18 with a COVID-19 diagnosis (WHO criteria) [23]. Patients with severe cognitive impairments or inability to give informed consent were excluded. Informed consent was requested after explaining the study procedures and objectives. The research team collected the following demographics, and clinical data for all the patients included: age, sex, symptoms’ onset, disease severity (WHO criteria) [24], hospital length of stay (LOS), intubation (in days), assistive ventilation type, comorbidity, clinical complications, and discharge setting destination (home, multidisciplinary rehabilitation, low-intensive rehabilitation). At 12 weeks from the symptoms’ onset, all patients had been contacted by the clinical research team to propose and plan an appointment for clinical evaluation.

All patients were assessed using the C19-YRS questionnaire [19]. The scale was completed by patients during a visit at the outpatient rehabilitation service or was administered by phone. The checklist guided the clinical interview and aimed to underline the persistent symptoms and their intensities concerning the pre-infection condition in a bio-psycho-social way. Moreover, it highlighted three different classes of PCS severity for each symptom (<3 = mild; 3–5 = moderate; 6–10 = severe) [25]. The clinical assessment through the C19-YRS application covered all the domains according to the International Classification of Functioning (ICF), thus investigating symptoms related to body functions and structures, limitations to activities, and participation restrictions considering personal and environmental factors. We selected 10 of the 22 items, the ones directly or indirectly measurable through validation. In the tests and questionnaires, thus we were concerned with: breathlessness at rest (BF1a), during dressing (BF1b) and during stairs (BF1c), fatigue (BF2), pain/discomfort (BF3), anxiety (BF4), depression (BF5), communication (A1), mobility (A2), personal care (A3), usual activities (A4), and global health (P1). Furthermore, a set of established outcome measures were used for investigating COVID-19 sequalae: (i) the 6 min walking test (6MWT) has been executed for investigating exercise tolerance [26]; (ii) the 5 time-sit-to-stand (5 time-STS) [27] to quantify the lower limb strength; (iii) the Montreal Cognitive Assessment (MoCA) for cognitive functions [28]; (iv) the Beck Anxiety Inventory (BAI) and Patient Health Questionnaire-9 (PHQ9) for investigating anxiety [29] and depressive symptoms [30]. The overall perceived HR-QoL has been detected by using the Short Form Health Survey (SF-12) in its mental and physical components [31]. All the evaluations have been performed by clinical researchers trained for the application of the afore-mentioned clinical tests.

Descriptive statistics (mean, standard deviation, and frequency) were calculated for all clinical parameters and C19-YRS items for ICU-hospitalized and Ward-hospitalized patients. Data distribution were verified through a Shapiro–Wilk test. Due to the skewness of data, the rank-sum test or the Chi-Square test was used for between-groups differences. C19-YRS outcomes have been examined considering frequency and intensity in the answers given. The scale’s internal consistency has been checked by Cronbach’s alpha coefficient, a magnitude index for the between-items coherence, where values of >0.70 represent high consistency. Correlations between what was revealed by the C19-YRS items to the correspondent test’s score (Table 1) have been calculated using the Spearman’s rho.

The magnitude scale proposed by Hopkins [32] was used to interpret the correlation coefficients: <0.1, trivial; 0.1–0.29, small; 0.30–0.49, moderate; 0.50–0.69, high; 0.70–0.90, very high; >0.90, almost perfect. The responsiveness of each C19-YRS item between 12 and 26 weeks was measured by distribution-based methods such as the effect size (ES) and standardized response mean (SRM) [33]. The ES, calculated for each item as the difference between 12- and 26-week scores divided by the pre-test standard deviation (SD), was classified as small (0.20–0.39), moderate (0.4–0.7), or large (>0.7). The SRM, that is, the difference between means divided by the standard deviation of the difference, represented small (0.2), moderate (0.5) or large (0.8) changes. The sample size calculation was based on Terwee’s methodology, in which at least 50 subjects are needed for analyzing the instrument’s construct validity [34]. The statistical significance was set at 0.05. STATA 13.1 (College Station, TX, USA: StataCorp LP) and MedCalc Statistical Software version 20.014 (MedCalc Software Ltd., Ostend, Belgium) were used for analysis.

## 3. Results

Seventy-nine patients (60.8% men, 66 years old) hospitalized for COVID-19 infection were included in this longitudinal study (Table 2). Among them, 66 patients returned to the hospital for the 26-week visit (13 patients declined the second clinical evaluation due to difficulties in reaching the hospital).

**Figure 1:**
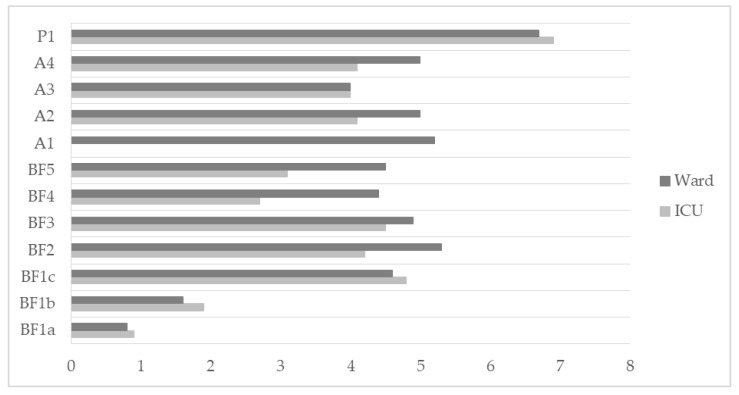
COVID-19 mean symptom intensity at 12 weeks in ICU- and Ward-admitted patients. Legend: BF1 = Breathlessness: (a) at rest, (b) dressing, (c) stairs; BF2 = Fatigue; BF3 = Pain/discomfort; BF4 = Anxiety; BF5 = Depression; A1 = Communication; A2 = Mobility; A3 = Personal Care; A4 = Usual Activities; P1 = Global Health.

Patients who were admitted to an intensive care unit (*n* = 49) were significantly younger (*p* < 0.001), with longer LOS (*p* < 0.01), a higher incidence of tracheostomy (*p* < 0.01), dysphagia (*p* < 0.01), and sepsis (*p* < 0.01). Moreover, they were more likely discharged to a multidisciplinary inpatient rehabilitation setting (*p* < 0.01) (Table 2). For patients who could not come to the 12-week visit, C19-YRS was administered by phone (n = 18). After 12 weeks, 88.5% reported fatigue, 83.3% breathlessness, 69.4% anxiety, 55.6% depression, and 44.9% pain, (Body Functions and Structures). Breathlessness was more present during highly demanding tasks, such as climbing stairs, with a C19-YRS reported intensity of 4.0 (2.7). Similarly, fatigue was rated with an intensity of 4.3 (2.9). Finally, anxiety and depression were scored with 2.2 (2.7) and 2.0 (2.7) intensity. Among Activities, reduced mobility was the most significant limitation highlighted (84.6%) after 12 weeks, with a significant influence on usual activities (75.7%) and personal care (47.4%). Half of the patients (55.8%) were retired, whereas 14.3% were still on sick leave because of COVID-19. However, no differences were highlighted between patients admitted or not to ICU (Table 3 and Figure 1).

Among the Body Functions and Structures items, the correlations were high between BF1 during stairs (rho −0.52; *p* < 0.01), BF2 (rho −0.50; *p* < 0.01), and the 6MWT or between BF4 (rho 0.52; *p* < 0.01) and BAI. Whereas, it was moderate between BF1 during stairs (rho 0.47; *p* < 0.01), BF2 (rho 0.46; *p* < 0.01) and 5 time-STS or between BF5 (rho 0.32; *p* < 0.01) and PHQ-9. The Activities items were highly correlated with the 6MWT (A2: rho 0.60; *p* < 0.01); A4: rho −0.54; *p* < 0.01) and moderately correlated with the 5 time-STS (A2: rho 0.40; *p* < 0.01; A3: rho 0.31; *p* < 0.01). Finally, the Global Health perception was correlated with MCS-12 (rho 0.25; *p* = 0.01) and PCS-12 (rho 0.39; *p* < 0.01). Further correlations were reported in Table 4. As reported by the Cronbach’s alpha (0.87), the internal consistency was high considering the overall scale.

At 26 weeks, except for anxiety, all the PCS symptoms were still present but significantly reduced. Physical symptoms such as breathlessness improved by 16%, fatigue by 19%, whereas the variation of psychological symptoms, such as anxiety (12%) and depression (7%), was less pronounced. Conversely, pain (+1%) was more represented evidencing a persistence over time. Among activities, a significant improvement in mobility (36%), personal care (22%), and usual activities (18%) were found, whereas communication became worse (+6%). The variation of intensities and the distribution-based measures of responsiveness were reported in Table 5.

**Figure 2:**
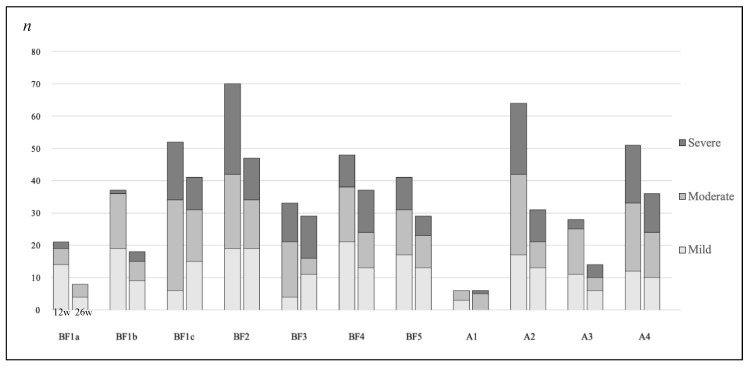
Changes of PCS severity phenotypes (mild, moderate, severe). Legend: BF1 = Breathlessness: (a) at rest, (b) dressing, (c) stairs; BF2 = Fatigue; BF3 = Pain/discomfort; BF4 = Anxiety; BF5 = Depression; A1 = Communication; A2 = Mobility; A3 = Personal Care; A4 = Usual Activities.

The C19-YRS had moderate responsiveness for breathlessness (ES 0.58 [0.21 to 0.94] during dressing, SRM −0.54 [−0.83 to −0.23] during stairs), fatigue (ES 0.56 [0.21 to 0.90] and SRM −0.54 [−0.79 to −0.26]), and mobility (ES 0.63 [0.29 to 0.99] and SRM 0.63 [−0.86 to −0.33]), which were the most ICF-related deviations reported in our sample. Finally, significant changes in PCS severity phenotypes (mild, moderate, and severe) were found at 26 weeks for all C19-YRS items assessed (Figure 2). For example, a reduction of the severe phenotype was found for BF1a, BF1c, BF2, BF5, A2, and A4. Conversely, this phenotype was more represented for BF1b, BF3, BF4, A1, and A3, revealing the possible fluctuation of PCS over time.

## 4. Discussion

This is the first study that applied the C19-YRS in a non-UK population to test its usability in a cohort of hospitalized patients, and the first study ever to report the responsiveness of the scale in PCS. This study demonstrated that this scale can be a valuable tool in detecting the multidimensional nature of Post-COVID Syndrome, characterized by physical, cognitive, and psychological symptoms that persist longer than 12 weeks from the acute infection [9,11]. Our sample reported persistent symptoms related to Body Functions and Structures, such as fatigue (88.5%), breathlessness (83.3%), pain (44.9%), anxiety (69.4%), and depression (55.6%), and Activities such as reduced mobility (84.6%) and usual activities (75.7%) after 12 weeks. These findings are consistent with previous studies that reported the persistence of symptoms in post-COVID patients [5,8,35,36], even though no clear conclusions have been drawn on the pathophysiology of this condition, the duration, and determinant factors. For example, our findings supported that COVID-19 severity or intensive care hospitalization do not directly influence the persistence of symptoms after 12 weeks [25,36]. No differences have been found between patients admitted to the ICU or Ward in our cohort. A possible explanation is that other factors, such as age, may have masked the influence of COVID severity on PCS. Our Ward patients were significantly older than ICU patients, and it has been established how age is negatively related to PCS [12,37].

After 26 weeks, the more common PCS physical symptoms, such as breathlessness and fatigue, were significantly less represented. Similarly, the severity of phenotypes was changed, with a small portion of patients with persistent severe symptoms. The symptoms’ entity may be related to several factors such as the extent of organ damage, the different recovery time required of each system, the persistence of chronic inflammation or immune response generation, post-intensive care syndrome, complications related to comorbidities or adverse effects caused by medications used [38]. Psychological distress was less pronounced in the whole sample, even if anxiety was still present and with a more severe phenotype. These findings might reflect different recovery profiles for physical and psychological functions after COVID-19, leading the hypothesis that long-term management of psychological well-being might be warranted in PCS. Indeed, various adverse neurocognitive and psychiatric outcomes after COVID-19 have been reported. Among psychiatric consequences of infection high rates of post-traumatic stress disorder, depression, anxiety, insomnia, and obsessive-compulsive symptomatology were reported [39,40]. The psychopathological outcome seems to be caused by the immune response to the virus itself and by psychological stressors such as social isolation, stigma, fear, uncertainty, and traumatic memories of illness [41]. We highlighted a significant improvement of mobility, personal care, and usual activities regarding Activities. However, for the personal care domain, people with a severe phenotype at 12 weeks still reported a similar limitation after three months with patients who reported further deterioration of this fundamental daily life activity.

The ability of the C19-YRS to sensitively encode all the aspects of the bio-psycho-social ICF model is particularly suitable for implementing tailored rehabilitation interventions for PCS in clinical settings. Indeed, the scale was designed by a multidisciplinary rehabilitation team [19], where specialists are trained to manage different aspects of care of post-COVID patients (i.e., respiratory physiotherapist, motor physiotherapist, clinical psychologist, neuropsychologist, speech therapist, dietitian) coordinated by the physical medicine and rehabilitation physician, and in close collaboration with the respiratory medicine and intensive care teams. The C19-YRS can also be used as a screening tool to identify specific post-COVID sequelae, and to refer to comprehensive and integrated rehabilitation pathways [42].

The construct validity of the C19-YRS was proved by the correlation between the C19-YRS items and a basket of validated outcome measures of exercise tolerance (6MWT), lower limb strength, and mobility (5 time-STS), anxiety (BAI), depression (PHQ-9), and HR-QoL (SF-12) [43]. The findings suggest that single item questions for each symptom in the C19-YRS have good correlation to well-validated full scales developed for those symptoms. This is an important finding supporting the use of a single condition-specific comprehensive outcome measure (C19-YRS) rather than burdening the patient with multiple symptom-specific measures [44]. Moreover, the internal consistency was high, as O’Connor et al. [17] reported in a mainly non-hospitalized COVID-19 cohort. The distribution-based measures highlighted that C19-YRS had moderate responsiveness between 12 and 26 weeks for the more represented symptoms (breathlessness and fatigue) and activity (mobility), confirming the hypothesis that it can be used as a valuable tool to monitor their evolution over time. The low responsiveness for the other domains can reflect the fluctuation of symptoms seen in PCS before [42].

This study has several limitations. Firstly, the relatively small sample size prevented us from making definitive conclusions. Secondly, this was an observational study where patients received different rehabilitation treatments (motor or respiratory rehabilitation, psychological counseling) and settings in the post-discharge phase (inpatient multidisciplinary or low-intensive rehabilitation, home) which can be considered a bias for detecting responsiveness. Thirdly, we included only hospitalized COVID-19 patients. For this reason, the results cannot be generalized to the entire post-COVID population. Fourthly, symptoms and their severity were self-reported by COVID-19 patients, leading to a possible degree of subjectivity in their reporting. Lastly, a cross-cultural translation of the Italian version of the C19-YRS has not been done so far.

## 5. Conclusions

The C19-YRS was a valuable patient-reported outcome for screening, assessing severity, and monitoring the persistence of symptoms after 12 and 26 weeks from SARS-CoV2 infection in a cohort of Italian hospitalized patients. The selected single items of the scale demonstrated good construct validity compared to other established scales, supporting the use of the scale to comprehensively capture the multisystem condition. The scale also helped identify multidimensional rehabilitation needs (physical and psychological) which can inform tailored intervention through a comprehensive biopsychosocial assessment. Further research on construct validity and responsiveness is needed with a larger sample and in non-hospitalized patients. Moreover, a cross-cultural translation of the Italian version of the C19-YRS is warranted.

## Figures and Tables

**Table 1:** C19-YRS items and related Outcome measures within a ICF framework. Abbreviations: 6MWT = 6-Minutes Walking Test; 30 secs-STS = 30 s Sit-To-Stand; 5 Time-STS = 5 Time-Sit-To-Stand; BAI = Beck Anxiety Inventory; PHQ-9 = Patient Health Questionnaire-9; MCS-12 = Mental Health Composite Scale—Short Form 12; PCS-12 = Physical Composite Scale—Short Form-12; MoCA = Montreal Cognitive Assessment.

Body Functions and Structures	
**C19-YRS Items**	**Outcome Measures**
BF1—Breathlessness (at rest, dressing, stairs)	6MWT, 5 Time-STS
BF2—Fatigue	6MWT, 5 Time-STS, BAI, PHQ-9, MCS-12, PCS-12
BF3—Pain/discomfort	
BF4—Anxiety	BAI
BF5—Depression	PHQ-9
**Activities**	
**C19-YRS Items**	**Outcome Measures**
A1—Communication	MoCA
A2—Mobility	6MWT, 5 Time-STS
A3—Personal Care	
A4—Usual Activities	
**Personal Factors**	
**C19-YRS Items**	**Outcome Measures**
P1—Global Health	MCS-12, PCS-12

**Table 2:** Demographic and clinical characteristics of COVID-19 patients admitted to ordinary wards and intensive care units. Abbreviations: ICU= Intensive Care Unit; CINM = Critical Illness Neuromyopathy; ARDS = Acute Respiratory Distress Syndrome; LOS = Length of Stay.

	ICU Patients	Ward Patients	Total	*p* Value
	(*n* = 42)	(*n* = 37)	(*n* = 79)	
Age, years	61 (11)	71 (12)	66 (13)	<0.001
Sex:				0.82
Men (%)	61.9%	59.5%	60.8%	
Women (%)	38.1%	40.5%	39.2%	
Obesity (%)	28.6%	11.1%	20.5%	0.06
Hypertension (%)	54.8%	66.7%	60.3%	0.28
Diabetes (%)	14.3%	25%	19.2%	0.23
COVID-19 severity:				0.01
Severe (%)	0%	13.5%	6.3%	
Critical (%)	100%	86.5%	93.7%	
LOS (days) in acute care	39 (21)	27 (17)	33 (20)	<0.01
Tracheostomy (%)	26.2%	0%	13.9%	<0.01
Pronation cycles (%)	31.7%	5.4%	19.2%	<0.01
CINM (%)	9.5%	0%	5.0%	0.05
Stroke (%)	0%	2.7%	1.2%	0.28
Dysphagia (%)	7.1%	0%	3.8%	0.01
Sepsis (%)	35.7%	8.1%	22.8%	<0.01
Acute renal failure (%)	7.1%	8.1%	7.6%	0.87
ARDS (%)	11.9%	16.2%	13.9%	0.58
Respiratory failure (%)	100%	97.3%	98.7%	0.28
Discharge setting:				<0.01
Home (%)	54.8%	59.5%	57.0%	
Low-intensive rehabilitation (%)	4.7%	27.0%	15.2%	
Multidisciplinary rehabilitation (%)	40.5%	13.5%	27.8%	
Employment:				0.23
Retired (%)	47.7%	65.7%	55.8%	
Not working (%)	19.0%	8.6%	14.3%	
Working (%)	33.3%	25.7%	29.9%	

**Table 3:** COVID-19 symptoms after 12 weeks. Abbreviations: ICU = Intensive Care Unit.

C19-YRS Items	ICU Patients	Ward Patients	Total	*p* Value
	(*n* = 42)	(*n* = 37)	(*n* = 79)	
**Body Functions and Structures**				
Breathlessness (%)	83.3	83.3	83.3	1.00
at rest (intensity)	0.8 (1.5)	0.7 (1.5)	0.7 (1.5)	0.49
dressing (intensity)	1.6 (1.8)	1.3 (1.7)	1.4 (1.8)	0.50
stairs (intensity)	3.9 (2.6)	4.1 (3.0)	4.0 (2.7)	0.85
Fatigue (%)	90.5	86.1	88.5	0.55
intensity	4.0 (2.5)	4.7 (3.2)	4.3 (2.9)	0.30
Pain/discomfort (%)	38.1	52.8	44.9	0.19
intensity	1.7 (2.9)	2.6 (3.0)	2.1 (2.9)	0.14
Anxiety (%)	61.9	69.4	65.4	0.48
intensity	1.6 (2.0)	2.9 (3.2)	2.2 (2.7)	0.12
Depression (%)	50	55.5	52.6	0.62
intensity	1.5 (2.3)	2.5 (3.1)	2.0 (2.7)	0.28
**Activities**				
Communication (%)	0%	16.7%	7.7%	<0.01
intensity	0 (0)	0.5 (1.3)	0.2 (0.9)	<0.01
Mobility (%)	88.1%	80.5%	84.6%	0.36
intensity	3.6 (2.5)	4.1 (3.4)	3.8 (2.9)	0.63
Personal Care (%)	42.8%	52.8%	47.4%	0.38
intensity	1.7 (2.5)	2.1 (3.1)	1.9 (2.8)	0.61
Usual Activities (%)	72.2%	79.4%	75.7%	0.48
intensity	3.0 (2.5)	3.9 (3.6)	3.4 (3.1)	0.35
**Personal Factors**				
Global Health intensity	6.9 (1.7)	6.7 (2.1)	6.8 (1.8)	0.64

**Table 4:** Correlations between the C19-YRS items and outcome measures. Abbreviations: 6MWT = 6-Minutes Walking Test; 5 Time-STS = 5 Time-Sit-To-Stand; BAI = Beck Anxiety Inventory; PHQ-9 = Patient Health Questionnaire-9; MCS-12 = Mental Health Composite Scale—Short Form 12; MoCA = Montreal Cognitive Assessment.

KERRYPNX	Body Functions and Structures		
**C19-YRS Items**	**Test**	**Rho**	***p*** **Value**
Breathlessness (dressing)	6MWT	−0.35	<0.01
	5 time-STS	0.33	<0.01
Breathlessness (stairs)	6MWT	−0.52	<0.01
	5 time-STS	0.47	<0.01
Fatigue	6MWT	−0.50	<0.01
	5 time-STS	0.46	<0.01
Pain/discomfort	6MWT	−0.24	0.02
	5 time-STS	0.28	0.02
	BAI	0.42	<0.01
	PHQ-9	0.38	<0.01
	MCS-12	−0.23	0.02
	PCS-12	−0.28	<0.01
Anxiety	BAI	0.52	<0.01
Depression	PHQ-9	0.32	<0.01
**Activities**			
**C19-YRS Items**	**Test**	**Rho**	***p*** **Value**
Mobility	6MWT	−0.60	<0.01
	5 time-STS	0.40	<0.01
Personal Care	6MWT	−0.52	<0.01
	5 time-STS	0.47	<0.01
Usual Activities	6MWT	−0.54	<0.01
**Personal Factors**			
**C19-YRS Items**	**Test**	**Rho**	***p*** **Value**
Global Health	MCS-12	−0.25	0.01
	PCS-12	0.39	<0.01

**Table 5:** Responsiveness of COVID-19 symptoms at 12 and 26 weeks. Abbreviations: ICU = Intensive Care Unit, M = Mean, SD = Standard Deviation, ES = effect size, SRM = standardized response means.

C19-YRS Items	Totals (*n* = 66)		*p* Value	SRM [CI 95%]	ES [CI 95%]
	12 Weeks	26 Weeks			
**Body Functions and Structures**					
Breathlessness (%)	83.3%	66.7%	0.02		
at rest (M, SD)	0.7 (1.5)	0.3 (1.1)	0.16	−0.25 [−0.45, 0.05]	0.37 [0.02, 0.72]
dressing (M, SD)	1.5 (1.8)	0.8 (1.7)	0.04	−0.33 [−0.59, −0.03]	0.58 [0.21, 0.94]
stairs (M, SD)	4.1 (2.8)	2.5 (2.8)	<0.01	−0.54 [−0.83, −0.23]	0.25 [−0.09, 0.59]
Fatigue (%)	87.9%	68.2%	0.04		
(M, SD)	4.3 (3.0)	2.7 (2.7)	<0.01	−0.54 [−0.79, −0,26]	0.56 [0.21, 0.90]
Pain/discomfort (%)	42.4%	43.9%	0.02		
(M, SD)	1.8 (2.7)	1.9 (2.8)	0.98	0.00 [−0.24, 0.23]	−0.01 [-0.35, 0.34]
Anxiety (%)	59.1%	47.0%	0.07		
(M, SD)	1.8 (2.5)	2.5 (3.0)	0.22	0.17 [−0.06, 0.40]	−0.22 [−0.56, 0.13]
Depression (%)	47.0%	39.4%	<0.01		
(M, SD)	1.8 (2.7)	1.6 (2.4)	0.68	−0.07 [−0.30, 0.15]	0.07 [−0.27, 0.41]
**Activities**					
Communication (%)	3.0%	9.1%	0.04		
(M, SD)	0.0 (0.2)	0.4 (1.4)	0.02	0.29 [0.16, 0.46]	−0.40 [−0.75, −0.06]
Mobility (%)	83.3%	47.0%	<0.01		
(M, SD)	3.8 (3.0)	1.9 (2.8)	<0.01	0.63 [−0.86, −0.33]	0.63 [0.29, 0.99]
Personal Care (%)	45.4%	22.7%	<0.01		
(M, SD)	1.8 (2.8)	0.8 (2.0)	0.02	−0.41 [−0.63, −0.15]	0.42 [0.07, 0.76]
Usual Activities (%)	71.7%	53.3%	<0.01		
(M, SD)	3.3 (3.2)	2.3 (2.8)	0.07	−0.32 [−0.57, −0.05]	0.33 [−0.03, 0.68]
**Personal Factors**					
Global Health					
(M, SD)	7.0 (1.7)	7.6 (1.8)	0.08	0.25 [−0.03, 0.49]	−0.30 [−0.65, 0.04]

## Data Availability

The research data used in this paper are publicly available at: doi:10.17632/wsmh5t4p3n.1.

## References

[1] Epicentro—L’Epidemiologia per la Sanità Pubblica. https://www.epicentro.iss.it/coronavirus/sars-cov-2-dashboard.

[2] Wu Z., McGoogan J.M. (2020). Characteristics of and Important Lessons from the Coronavirus Disease 2019 (COVID-19) Outbreak in China: Summary of a Report of 72 314 Cases from the Chinese Center for Disease Control and Prevention. JAMA.

[3] Boldrini P., Bernetti A., Fiore P., SIMFER Executive Committee, SIMFER Committee for International Affairs (2020). Impact of COVID-19 outbreak on rehabilitation services and Physical and Rehabilitation Medicine physicians’ activities in Italy. An official document of the Italian PRM Society (SIMFER). Eur. J. Phys. Rehabil. Med..

[4] Agostini F., Mangone M., Ruiu P., Paolucci T., Santilli V., Bernetti A. (2021). Rehabilitation setting during and after COVID-19: An overview on recommendations. J. Rehabil. Med..

[5] Malik P., Patel K., Pinto C., Jaiswal R., Tirupathi R., Pillai S., Patel U. (2022). Post-acute COVID-19 syndrome (PCS) and health-related quality of life (HRQoL)-A systematic review and meta-analysis. J. Med. Virol..

[6] Lopez-Leon S., Wegman-Ostrosky T., Perelman C., Sepulveda R., Rebolledo P.A., Cuapio A., Villapol S. (2021). More than 50 long-term effects of COVID-19: A systematic review and meta-analysis. Sci. Rep..

[7] Davis H.E., Assaf G.S., McCorkell L., Wei H., Low R.J., Re’em Y., Redfield S., Austin J.P., Akrami A. (2021). Characterizing long COVID in an international cohort: 7 months of symptoms and their impact. EClinicalMedicine.

[8] Carfì A., Bernabei R., Landi F. (2020). Gemelli against COVID-19 Post-Acute Care Study Group. Persistent Symptoms in Patients after Acute COVID-19. JAMA.

[9] Halpin S.J., McIvor C., Whyatt G., Adams A., Harvey O., McLean L., Walshaw C., Kemp S., Corrado J., Singh R. (2021). Postdischarge symptoms and rehabilitation needs in survivors of COVID-19 infection: A cross-sectional evaluation. J. Med. Virol..

[10] Nalbandian A., Sehgal K., Gupta A., Madhavan M.V., McGroder C., Stevens J.S., Cook J.R., Nordvig A.S., Shalev D. (2021). Post-acute COVID-19 syndrome. Nat. Med..

[11] Yong S.J. (2021). Long COVID or post-COVID-19 syndrome: Putative pathophysiology, risk factors, and treatments. Infect. Dis..

[12] Sudre C.H., Murray B., Varsavsky T., Graham M.S., Penfold R.S., Bowyer R.C., Pujol J.C., Klaser K., Antonelli M., Canas L.S. (2021). Attributes and predictors of long COVID. Nat. Med..

[13] Shah W., Hillman T., Playford E.D., Hishmeh L. (2021). Managing the long term effects of COVID-19: Summary of NICE, SIGN, and RCGP rapid guideline. BMJ.

[14] Webber S.C., Tittlemier B.J., Loewen H.J. (2021). Apparent Discordance between the Epidemiology of COVID-19 and Recommended Outcomes and Treatments: A Scoping Review. Phys. Ther..

[15] Rooney S., Webster A., Paul L. (2020). Systematic Review of Changes and Recovery in Physical Function and Fitness after Severe Acute Respiratory Syndrome-Related Coronavirus Infection: Implications for COVID-19 Rehabilitation. Phys. Ther..

[16] Sivan M., Halpin S., Hollingworth L., Snook N., Hickman K., Clifton I.J. (2020). Development of an integrated rehabilitation pathway for individuals recovering from COVID-19 in the community. J. Rehabil. Med..

[17] O’Connor R.J., Preston N., Parkin A., Makower S., Ross D., Gee J., Halpin S.J., Horton M., Sivan M. (2021). The COVID-19 Yorkshire Rehabilitation Scale (C19-YRS): Application and psychometric analysis in a post-COVID-19 syndrome cohort. J. Med. Virol.

[18] Sivan M., Halpin S., Gee J., Makower S., Parkin A., Ross D., Horton M., O’Connor R. (2021). The self-report version and digital format of the COVID-19 Yorkshire Rehabilitation Scale (C19-YRS) for Long COVID or Post-COVID syndrome assessment and monitoring. Adv. Clin. Neurosci. Rehabil..

[19] Sivan M., Halpin S.J., Gee J. (2020). Assessing long-term rehabilitation needs in COVID-19 survivors using a telephone screening tool (C19-YRS tool). ACNR.

[20] NHS England (2021). National Guidance for Post-COVID Syndrome Assessment Clinics.

[21] Sivan M., Taylor S. (2020). NICE guideline on long COVID. BMJ..

[22] WHO (2021). Support for Rehabilitation: Self-Management after COVID-19-Related Illness.

[23] World Health Organization (2020). Diagnostic Testing for SARS-CoV-2: Interim Guidance.

[24] Agarwal A., Rochwerg B., Siemieniuk R.A., Agoritsas T., Lamontagne F., Askie L., Lytvyn L., Leo Y.S., Macdonald H., Zeng L. (2020). A living WHO guideline on drugs for COVID-19. BMJ.

[25] Sivan M., Parkin A., Makower S., Greenwood D.C. (2022). Post-COVID syndrome symptoms, functional disability, and clinical severity phenotypes in hospitalized and nonhospitalized individuals: A cross-sectional evaluation from a community COVID rehabilitation service. J. Med. Virol..

[26] ATS Committee on Proficiency Standards for Clinical Pulmonary Function Laboratories (2002). ATS statement: Guidelines for the six-minute walk test. Am. J. Respir. Crit. Care Med..

[27] Bohannon R.W., Bubela D.J., Magasi S.R., Wang Y.C., Gershon R.C. (2010). Sit-to-stand test: Performance and determinants across the age-span. Isokinet. Exerc. Sci..

[28] Nasreddine Z.S., Phillips N.A., Bédirian V., Charbonneau S., Whitehead V., Collin I., Cummings J.L., Chertkow H. (2005). The Montreal Cognitive Assessment, MoCA: A brief screening tool for mild cognitive impairment. J. Am. Geriatr. Soc..

[29] Beck A.T., Epstein N., Brown G., Steer R.A. (1988). An inventory for measuring clinical anxiety: Psychometric properties. J. Consult. Clin. Psychol..

[30] Kroenke K., Spitzer R.L., Williams J.B. (2001). The PHQ-9: Validity of a brief depression severity measure. J. Gen. Intern. Med..

[31] Cheak-Zamora N.C., Wyrwich K.W., McBride T.D. (2009). Reliability and validity of the SF-12v2 in the medical expenditure panel survey. Qual. Life Res..

[32] Hopkins W.G. (2000). Measures of reliability in sports medicine and science. Sports Med..

[33] Cohen J., Hillsdale N.J. (1988). Statistical Power Analysis for the Behavioral Sciences.

[34] Terwee C.B., Bot S.D., de Boer M.R., van der Windt D.A., Knol D.L., Dekker J., Bouter L.M., de Vet H.C. (2007). Quality criteria were proposed for measurement properties of health status questionnaires. J. Clin. Epidemiol..

[35] Halpin S., O’Connor R., Sivan M. (2021). Long COVID and chronic COVID syndromes. J. Med. Virol..

[36] Garrigues E., Janvier P., Kherabi Y., Le Bot A., Hamon A., Gouze H., Doucet L., Berkani S., Oliosi E., Mallart E. (2020). Post-discharge persistent symptoms and health-related quality of life after hospitalization for COVID-19. J. Infect..

[37] Jacobs L.G., Gourna Paleoudis E., Lesky-Di Bari D., Nyirenda T., Friedman T., Gupta A., Rasouli L., Zetkulic M., Balani B., Ogedegbe C. (2020). Persistence of symptoms and quality of life at 35 days after hospitalization for COVID-19 infection. PLoS ONE.

[38] Raveendran A.V., Jayadevan R., Sashidharan S. (2021). Long COVID: An overview. Diabetes Metab Syndr..

[39] Rogers J.P., Chesney E., Oliver D., Pollak T.A., McGuire P., Fusar-Poli P., Zandi M.S., Lewis G., David A.S. (2020). Psychiatric and neur;psychiatric presentations associated with severe coronavirus infections: A systematic review and meta-analysis with comparison to the COVID-19 pandemic. Lancet Psychiatry.

[40] Mazza M.G., De Lorenzo R., Conte C., Poletti S., Vai B., Bollettini I., Melloni E.M., Furlan R., Ciceri F., Rovere-Querini P. (2020). Anxiety and depression in COVID-19 survivors: Role of inflammatory and clinical predictors. Brain Behav. Immun..

[41] Carvalho P.M.M., Moreira M.M., de Oliveira M.N.A., Landim J.M.M., Neto M.L.R. (2020). The psychiatric impact of the novel coronavirus outbreak. Psychiatry Res..

[42] Parkin A., Davison J., Tarrant R., Ross D., Halpin S., Simms A., Salman R., Sivan M. (2021). A Multidisciplinary NHS COVID-19 Service to Manage Post-COVID-19 Syndrome in the Community. J. Prim. Care Community Health.

[43] Patel K., Straudi S., Yee Sien N., Fayed N., Melvin J.L., Sivan M. (2020). Applying the WHO ICF Framework to the Outcome Measures Used in the Evaluation of Long-Term Clinical Outcomes in Coronavirus Outbreaks. Int. J. Environ. Res. Public Health..

[44] Sivan M., Wright S., Hughes S., Calvert M. (2022). Using condition specific patient reported outcome measures for long COVID. BMJ.

